# Food and water security issues in Russia III: food- and waterborne diseases in the Russian Arctic, Siberia and the Far East, 2000–2011

**DOI:** 10.3402/ijch.v72i0.21856

**Published:** 2013-12-09

**Authors:** Alexey A. Dudarev, Vitaliy M. Dorofeyev, Eugenia V. Dushkina, Pavel R. Alloyarov, Valery S. Chupakhin, Yuliya N. Sladkova, Tatjana A. Kolesnikova, Kirill B. Fridman, Lena Maria Nilsson, Birgitta Evengard

**Affiliations:** 1 Northwest Public Health Research Center, St. Petersburg, Russia; 2 Dubna City Hospital, Moscow Oblast, Russia; 3 Division of Nutritional Research, Department of Public Health and Clinical Medicine, Umeå University, Umeå, Sweden; 4 Arcum – Arctic Research Centre, Umeå University, Umeå, Sweden; 5 Division of Infectious Diseases, Department of Clinical Microbiology, Umeå University, Umeå, Sweden

**Keywords:** infections, parasites, food- and waterborne diseases, Russian Arctic

## Abstract

**Background:**

The food- and waterborne disease situation in Russia requires special attention. Poor quality of centralized water supplies and sewage systems, biological and chemical contamination of drinking water, as well as contamination of food products, promote widespread infectious diseases, significantly exceeding nationwide rates in the population living in the two-thirds of Russian northern territories.

**Objectives:**

The general aim was to assess the levels of food- and waterborne diseases in selected regions of Russian Arctic, Siberia and the Far East (for the period 2000–2011), and to compare disease levels among regions and with national levels in Russia.

**Study design and methods:**

This study is the first comparative assessment of the morbidity in these fields of the population of 18 selected regions of Russian Arctic, Siberia and the Far East, using official statistical sources. The incidences of infectious and parasitic food- and waterborne diseases among the general population (including indigenous peoples) have been analyzed in selected regions (per 100,000 of population, averaged for 2000–2011).

**Results:**

Among compulsory registered infectious and parasitic diseases, there were high rates and widespread incidences in selected regions of shigellosis, yersiniosis, hepatitis A, tularaemia, giardiasis, enterobiasis, ascariasis, diphyllobothriasis, opistorchiasis, echinococcosis and trichinellosis.

**Conclusion:**

Incidences of infectious and parasitic food- and waterborne diseases in the general population of selected regions of the Russian Arctic, Siberia and the Far East (2000–2011) are alarmingly high. Parallel solutions must be on the agenda, including improvement of sanitary conditions of cities and settlements in the regions, modernization of the water supply and of the sewage system. Provision and monitoring of the quality of the drinking water, a reform of the general healthcare system and the epidemiological surveillance (including gender-divided statistics), enhancement of laboratory diagnostics and the introduction of preventive actions are urgently needed.

Six percent of the global burden of diseases is related to water (1), and from an Arctic perspective, food- and water borne diseases have recently been promoted as an indicator of interest in trans-Arctic monitoring (2). The situation in northern Russia requires special attention as, so far, few reports have been published on the incidence and prevalence of these infections. With climate change, a number of these diagnoses may potentially increase, and it is of value to have baseline data and knowledge of the current monitoring system. According to data from the ministerial department of sanitary-epidemiological surveillance (now *Rospotrebnadzor*), the poor quality of drinking water is the main cause of mass outbreaks of gastro-intestinal infectious diseases, including hepatitis A. In Russia, the number of outbreaks and sufferers from 2005 to 2007 were as follows: 62 outbreaks and 5,045 sufferers in 2005, 77 outbreaks and 2,381 sufferers in 2006, 52 outbreaks and 1,552 sufferers in 2007, with 32 of the outbreaks caused by the consumption of centralized drinking water (3).

Poor sanitation and unsatisfactory hygienic conditions in many of the northern settlements, crowded habitations, lack of safe drinking water in a majority of settlements, lack of a centralized water supply, sewage systems, organized waste handling and promote spread of infections. Two-thirds of Russian northern territories report a high incidence of bacterial dysentery, exceeding national levels, with the most unfavourable situation in Yakutia, Taimyr and Chukotka. For many years, the supply of good quality drinking water to the northern population has been a concern (4, 5).

Studies in Krasnoyarsk Kraj performed by scientists from Krasnoyarsk medical university showed that microbial contamination of food products increases the incidence in society of salmonellosis and shigellosis in outbreaks (6). These results are concurrent with findings of scientists from the Yakutian medical university, who detected a direct correlation between incidence of dysentery, salmonellosis and other intestinal infections, on the one hand, and the quality of drinking water and food products in Yakutsk city on the other (7). The direct association of giardiasis incidence in Yakutia with content of cysts in drinking water was confirmed by the regional Centre for Hygiene and Epidemiology in Yakutsk City (8).

## Objectives

The general aim of this study was to gather official data on the incidence of infectious and parasitic food- and waterborne diseases in regions of the Russian Arctic, Siberia and the Far East (from 2000 to 2011), and compare these with national levels.

## Study design and methods

A total of 18 regions of the Russian north, Siberia and the Far East have been included in the study, and the following official statistical data sources were used (for 2000–2011): the Federal “Social-Hygienic Monitoring” system, which contains data on infectious and parasitic food- and waterborne diseases in the selected regions.

Data on the incidence of infectious and parasitic food- and waterborne diseases among the general population (including indigenous) has been analyzed in the selected regions in northern Russia per 100,000 population, averaged for 2000–2011.

The Federal Social Hygiene Monitoring system collects data annually by compulsory reporting [“Information on infectious and parasitic diseases” (report form #2)] from all regions of the Russian Federation. Regional Centres for Hygiene and Epidemiology (*Rospotrebnadzor*) submit these data from regional reports [“Journals of registration of infectious diseases” (Form #060/u)] under “Special notifications about infectious diseases.” These notifications are prepared by physicians of all healthcare units of all agencies, in cases that are diagnosed as, or assumed to be, matters of infection or food poisoning, regardless of the circumstances surrounding patient contact (e.g. seeking treatment, being examined, or participating in a study in a hospital).

Morbidity data on Taimyr AO, Evenki AO and Koryak AO have been unavailable since 2007 due to the agglomeration of several regions of Russian Federation. Taymyr and Evenki AO were included in Krasnoyarsk region and Koryak AO became part of Kamchatka region.

Health statistics on indigenous people is difficult to obtain and is not reliable. Since 2002, this type of statistic is inaccessible (from official sources) as ethnicity was totally excluded from any statistical accounting and reporting. There are some data on aboriginal peoples’ health for 4 regions [Murmansk Oblast, Nenets AO, Taymyr AO and Chukotka AO could be found in the 2004 AMAP report (9)], but these are scarcely associated with food and water security.

In addition, most statistics in Russia contain no information on gender.

### Registration of infectious and parasitic food- and waterborne diseases in Russia

In the process of registration of infectious and parasitic diseases in Russia, the diagnostic capacities of hospitals are the weakest elements. According to WHO, in countries where the diagnoses listed in section A09 “Diarrhoea and gastroenteritis of presumed infectious origin” have not been verified, these should be attributed to diseases of non-infectious origin, and thus, should be classified as K52.9 “Non-infectious gastroenteritis and colitis, unspecified.” However, because of instructions from the Russian Ministry of Health Care and “due to unstable epidemiological situation in the country,” the diseases listed in section A09 are classified as intestinal infections. This means that the alternative subdivision K52.9 should not be used in Russia without a bacteriological examination of the patient. In 2000–2011, the portion of unidentified intestinal infections averaged 64% (in selected regions, 51–90%) of the total number of cases of intestinal infectious diseases. There is a tendency for an increase of unidentified (and unverified by a laboratory) intestinal infections during the last decade. These facts must be taken into account in international comparisons of intestinal infections.

The list of compulsory registered food- and waterborne specific infectious and parasitic diseases contain the following:

- *Bacterial*: typhoid fever, paratyphoid fever, shigellosis due to *Shigella sonnei*, shigellosis due to *Shigella flexneri*, intestinal *Escherichia coli*, *Campylobacter enteritis*, enteritis due to *Yersinia enterocolitica*;
- *Viral*: rotavirus enteritis, acute gastroenteropathy due to Norwalk agent, acute hepatitis A;
- *Zoonoses*: tularaemia, brucellosis, leptospirosis;
- *Protozoa*: aiardiasis, cryptosporidiosis, toxoplasmosis, amoebiasis;
- *Helminthiases*: ascariasis, trichuriasis, enterobiasis, trichinellosis, toxocariasis, *Taenia saginata* infection, *Taenia solium* infection, hymenolepiasis, diphyllobothriasis, echinococcosis, opisthorchiasis, clonorchiasis.

## Results

Specified food- and waterborne bacterial diseases (laboratory-verified) are presented in Table I.

**Table I:** Reported food- and waterborne bacterial diseases

Bacterial infections									

	Years	Typhoid	Paratyph	*Salmonella*	*Shigella sonnei*	*Shigella flexneri*	*Escherichia coli*	Yersinia	Campylobacter
Russian Federation	2002–11	0.097±0.01	0.013±0	34.8±0.2	14.6±0.1	16.5±0.1	11.5±0.1	1.88±0	0.36±0.02
Murmansk Oblast	2000–11	0.009±0.03	0.01±0.03	46.7±2.3	8.8±1	12.1±1.2	6.2±0.8	5.27±0.8	3.67±0.65
Karelia Republic	2000–11	0.012±0.04	0.011±0.04	50.2±2.7	32.5±2.2	29±2	1.6±0.5	3.12±0.7	0.3±0.21
Arkhangelsk Oblast	2000–11	0.066±0.06	0.006±0.02	50.7±2	29.3±1.5	17.8±1.2	20.5±1.3	13.5±1	0.04±0.06
Nenets AO	2000–11	0	0	43.7±10.2	20.6±7	3.7±3	1.6±1.9	3.72±3	0
Komi Republic	2000–11	0.031±0.06	0.025±0.05	45.9±2.2	14.8±1.2	17.7±1.3	6.8±0.8	0.66±0.3	0
Yamalo-Nenets AO	2000–11	0.048±0.1	0.016±0.05	45.9±3	17.1±1.8	19.1±1.9	9.2±1.3	1.53±0.5	0.02±0.05
Khanty-Mansi AO	2000–11	0.285±0.14	0.018±0.03	75.1±2.3	12.5±0.9	12.3±0.9	17.9±1.1	5.63±0.6	2.51±0.41
Taymyr AO	2000–06	0.364±0.98	0.72±1.38	30.1±8.9	32.3±9.2	94.4±15.7	nd	3.34±3	0
Evenki AO	2000–06	0	0	10.1±7.6	7.9±6.7	39.1±15	nd	0	0
Sakha Republic	2000–11	0.322±0.18	0	51.2±2.3	20.6±1.5	37.6±2	7.8±0.9	0.18±0.1	0
Magadan Oblast	2000–11	0	0	67.3±6.2	13.3±2.8	14±2.8	4±1.5	1.1±0.8	0
Koryak AO	2000–06	0	0	10.9±6.8	5.2±4.7	44.1±13.7	nd	0.71±1.7	0
Chukotka AO	2000–11	0	0	35.5±8.3	32.2±7.9	93.2±13.4	25.5±7	1.57±1.7	0
Kamchatka Kraj	2000–11	0.024±0.08	0	51.3±3.8	7±1.4	10.5±1.7	7.5±1.5	1.39±0.6	6.52±1.37
Sakhalin Oblast	2000–11	0.029±0.07	0	24.7±2.2	19.4±1.9	54.6±3.2	1.4±0.5	17.5±1.8	3.04±0.76
Khabarovsk Kraj	2000–11	0.023±0.04	0.006±0.02	43.2±1.7	9.6±0.8	32.1±1.5	2.1±0.4	2.05±0.4	0
Primorsky Kraj	2000–11	0.116±0.08	0.021±0.03	38±1.4	15.9±0.9	26.7±1.1	11.9±0.8	4.27±0.5	0
Amur Oblast	2000–11	0.042±0.07	0.009±0.03	38.6±2.1	24.2±1.7	43.3±2.2	14.6±1.3	1.71±0.4	0.02±0.04

Average incidence per 100,000 per year.

Typhoid – typhoid fever (A01.0). Data 2000–11 in Russian Federation; 2001–06 in Koryak AO.

Paratyph – paratyphoid fever (A01.1. 2. 3. 4). Data 2000–11 in Russian Federation; 2001–06 in Koryak AO.

*Salmonella* – salmonella infections (A01–A02).

*Shigella sonnei* – shigellosis (A03.3).

*Shigella flexneri* – shigellosis (A03.1).

*Escherichia coli* – intestinal *Escherichia coli* infections (A04.0. 1. 2. 3. 4). Data 2009–2011.

Yersinia – enteritis due to *Yersinia enterocolitica* (A04.6).

Campylobacter – *Campylobacter enteritis* (A04.5). Data 2000–11 in Russian Federation; 2001–06 in Koryak AO.

The incidence of *typhoid* fever (Table I) in the study regions was sporadic, sometimes due to “imported” cases. The total number of typhoid cases in Russia (2000–2011) was 1,670, of which 147 were registered in the northern regions studied as follows:

Murmansk Oblast: 10 cases distributed in several years,

Khanty-Mansi AO: 48 cases (17 cases in 2000),

Primorsky Kraj: 29 cases.

There was an outbreak of typhoid in Yakutia in 2002 when 37 people fell ill sick due to an accidental contamination of centralized drinking water supply system by sewage water. Paratyphoid fever is a very rare disease in selected regions. Between 2000 and 2011, a total of 19 cases (of 210 registered in Russia) were registered in 10 of the 18 regions; the highest rate was reported from Taymir, but it was only 2 cases in 2005, which is 0.72 per 100,000 averaged for 12 years.

The number of *Salmonella* infections in the regions studied was generally higher than in Russia (35 per 100,000) with a maximum in Magadan Oblast (67 per 100,000) and Khanty-Mansi AO (75 per 100,000) while the lowest levels were recorded in Evenki and Korjak AO (10–11 per 100,000). Among all identified salmonella strains, serogroup D represented from 66 to 100%.

In most of the regions, up to 99% of verified *shigellosis* dysentery are *S. sonnei* and *S. flexneri*. On average, statistically significant prevalence of *S. sonnei* was noted in Arkhangelsk Oblast and Nenets AO, while significant prevalence of *S. flexneri* was registered in Murmansk Oblast, Yakutia, Chukotka, Kamchatka, Sakhalin, Khabarovsk Kraj, Primorsky Kraj, Amur Oblast. A high number of cases of *S. sonnei*, compared to the nationwide Russian level (14.6 cases), was registered in Karelia, Arkhangelsk Oblast, Chukotka (about 2 times higher), while in Murmansk Oblast, Evenki AO, Korjak AO, Kamchatka and Khabarovsk Kraj the prevalence of *S. sonnei* was 5–10 cases per 100,000. A very high incidence of *S. flexneri* dysentery was reported in a majority of the selected regions. Taymir and Chukotka had extremely high incidences, with the latter showing 6 times higher than the Russian national average (16.5 cases). Only in Nenets AO was the disease rate actually lower (3.7 cases) than the national level.

The numbers of *Escherichia coli* infections were not high, as a rule, except for in Arkhangelsk Oblast, Khanty-Mansi AO and Chukotka, where the rate was about double the average level for Russia. The incidence of *Y. enterocolitica* was extremely high in Arkhangelsk Oblast (7 times as high as the Russian average) and Sakhalin Oblast (9 times higher). Of 6,102 cases of *C. enteritis* in Russia during the observation period, 1,317 cases were reported from the selected regions but in 10 of 18 regions, Campylobacter has not been registered, and in 4 regions the diagnosis was very rare. The exceptions that exceeded the national level by 7–18 times were Murmansk Oblast (371 cases), Khanty-Mansi AO (455 cases), Kamchatka (268 cases) and Sakhalin (189 cases).

Specified food- and waterborne viral and zoonotic diseases (laboratory-verified) are presented in Table II. *Rotaviral enteritis* frequently occurred in 8 regions exceeding the national average by 2–3 times. Of 9,579 cases of *Norwalk gastroenteropathy* in Russia (2009–2011), 1,762 cases were from these northern regions where the levels, however, differed. In Khanty-Mansi AO (977 cases totally) and Chukotka (41 cases), the rate was about 10 times greater compared to the average for Russia; the number of cases in Yamalo-Nenets AO was 120, with 106 in Kamchatka, and 243 cases in Khabarovsk Kraj in the course of 3 years. This infection is increasing in the whole country. *Hepatitis A* rates are rapidly increasing in Russia and particularly in the selected regions; very high levels (50–100 cases per 100,000) have been reported from Karelia, Evenki AO, Chukotka, Primorsky Kraj and Khabarovsky Kraj.

**Table II:** Reported food- and waterborne viral and zoonotic diseases

	Viral infections				Zoonoses		
		
	Years	Rotaviral enteritis	Norwalk	Hepatitis A	Tularaemia	Brucellosis	Leptospirosis
Russian Federation	2002–11	35±0.2	2.2±0	27±0.2	0.1±0	0.32±0	0.64±0
Murmansk Oblast	2000–11	83.8±3.1	0.54±0.2	23.4±1.6	0.02±0	0	0.32±0.2
Karelia Republic	2000–11	66.6±3.1	3.16±0.7	58.4±2.9	0.02±0.1	0.05±0.1	0.16±0.2
Arkhangelsk Oblast	2000–11	52.6±2	1.84±0.4	19.7±1.2	0.68±0.2	0.01±0	0.94±0.3
Nenets AO	2000–11	9.1±4.7	0	11.1±5.1	0.8±1.4	0	0.6±1.2
Komi Republic	2000–11	46.1±2.2	0.9±0.3	6.2±0.8	0.02±0	0	0.03±0.1
Yamalo-Nenets AO	2000–11	73.1±3.7	7.47±1.2	10.5±1.4	0.03±0.1	0.09±0.1	0.02±0.1
Khanty-Mansi AO	2000–11	106±2.7	21.3±1.2	11.8±0.9	0.14±0.1	0.04±0.1	0.02±0
Taymyr AO	2000–06	0	nd	20.8±7.4	7.62±4.5	1.05±1.7	0
Evenki AO	2000–06	0	nd	97.6±23.7	0.81±2.2	0	0
Sakha Republic	2000–11	38.7±2	0	25.6±1.6	0.01±0	0.14±0.1	0
Magadan Oblast	2000–11	91.8±7.3	7.75±2.1	12.6±2.7	0	0.09±0.2	0
Koryak AO	2000–06	9.8±6.5	nd	10.5±6.7	0	0	0
Chukotka AO	2000–11	75±12	27.1±7.2	49±9.7	0	0	0
Kamchatka Kraj	2000–11	91.9±5.1	10.8±1.8	9.1±1.6	0	0	0
Sakhalin Oblast	2000–11	121.5±4.8	1.57±0.5	19.2±1.9	0.08±0.1	0	0.16±0.2
Khabarovsk Kraj	2000–11	46.7±1.8	5.86±0.6	32.5±1.5	0.01±0	0.03±0	0.56±0.2
Primorsky Kraj	2000–11	48.9±1.6	0.39±0.1	57.7±1.7	0	0.08±0.1	0.11±0.1
Amur Oblast	2000–11	10.9±1.1	1±0.3	52.2±2.4	0.01±0	0.23±0.2	0

Average incidence per 100,000 per year.

Rotaviral enteritis – (A08.0).

Norwalk – acute gastroenteropathy due to Norwalk agent. Data 2009–2011.

Hepatitis A – acute Hepatitis A (B15).

Tularemia – (A21).

Brucellosis – (A23).

Leptospirosis – (A27).

Zoonoses in the Arctic, Siberia and the Far East are of particular concern as these regions are characterized by having some particular foci. Of 1,711 cases of *Tularemia* registered in Russia (2000–2011), 171 cases were registered in selected regions: in Arkhangelsk Oblast with 102 cases, in Khanty-Mansi AO, 24 cases, and 22 cases (7.2 per 100,000) in Taymir.

Tularemia is still very threatening despite the efforts of immunization. This disease is registered all over Siberia, particularly in its western part including the so-called oil-and-gas regions. Periods of low incidence of tularemia are replaced by periods of high numbers when 10s or even hundreds of people are contaminated in some regions, as in Taymir in 1991, Tyumen Oblast in 1981, 1983 and 1985, or in Novosibirsk Oblast and Omsk Oblast in 1996 (10).

Of 5,437 cases of *Brucellosis* registered in the Russian Federation (2000–2011), 91 cases were registered in the northern regions, 16 in Yakutia, 20 cases in Primorsky Kraj and 26 Amur Oblast – 26 cases. Taymir (only 3 cases) have the highest incidence (1.05 per 100,000). In the far northern regions of central Siberia, there are brucellosis foci in reindeer. Up to 60% of reindeer and 23% of the human population (representing 41.4% of herders, 10% of veterinarians and 14.3% of hunters) are infected. About 30% of human brucellosis cases are caused by consumption of meat that is not properly prepared (11).

Of 11,101 cases of *Leptospirosis* registered in Russia (2000–2011), 343 cases were registered in the selected regions (34 cases in Murmansk Oblast, 145 cases in Arkhangelsk Oblast, 99 cases in Khabarovsky Kraj, and 28 cases in Primorsky Kraj), with no registration of this disease in 8 regions. There is a tendency towards “urbanization” of leptospirosis, with the incidence rate among urban populations reaching 50–95% (in different regions), which is associated with infection of domesticated animals (dogs, pigs, cattle). The clinical symptoms of leptospirosis are also becoming increasingly severe, with an increase in mortality (10).

Specified food- and waterborne protozoa diseases (laboratory-verified) are presented in Table III. *Giardiasis* is the most common and widely spread protozoan infection in selected regions. The majority of the regions are characterized by high rates of giardiasis, with levels in some regions 3–4 times greater than the national rate. The regions with the highest incidence (more than 320 cases per 100,000) are Nenets AO and Magadan Oblast. *Cryptosporidiosis* is a very rare disease in Russia. Of 359 cases of cryptosporidiosis registered in Russia (2006–2011), 35 cases were registered in only 3 of the 18 selected regions (14 cases in Murmansk Oblast, 7 in Arkhangelsk Oblast, and 14 cases in Khanty-Mansi AO), while in 15 regions, this disease has not been registered. Of 10,090 cases of *toxoplasmosis* registered in Russia (2000–2011), 1,310 cases were registered in the selected regions (92 cases in Karelia, 714 cases in Arkhangelsk Oblast, 90 cases in Yamalo-Nenets AO, 61 cases in Khanty-Mansi AO, 306 in Yakutia, and 21 cases in Primorsky Kraj, with no registration of the disease in 5 of the regions. *Amoebiasis* is almost non-existent. Of 761 cases of amoebiasis registered (2009–2011), 26 cases were registered in only 5 of the 18 selected regions (3 cases in Murmansk Oblast, 1 case in Arkhangelsk Oblast, 3 cases in Komi Republic, and 17 cases in Khanty-Mansi AO), while in the 13 remaining selected regions, this disease has not been registered.

**Table III:** Reported food- and waterborne protozoal diseases and helminthiasis

	Protozoa					Helminthiases			
		
	Years	Giardiasis	Crypto sporidiosis	Toxoplasmosis	Amoebiasis	Ascariasis	Trichuriasis	Enterobiasis	Trichinellosis
Russian Federation	2002–11	77.5±0.3	0.04±0.01	0.58±0	0.18±0.01	40.6±0.2	1.24±0	316.4±0.5	0.23±0
Murmansk Oblast	2000–11	170.1±4.4	0.28±0.18	0.02±0	0.12±0.12	36.9±2.1	0.41±0.2	213.4±5	0
Karelia Republic	2000–11	76.8±3.3	0	1.11±0.4	0	17.4±1.6	0.14±0.1	348.5±7.1	0.02±0.1
Arkhangelsk Oblast	2000–11	97.1±2.7	0.09±0.08	4.62±0.6	0.03±0.05	19.3±1.2	0.16±0.1	652±7.1	0.01±0
Nenets AO	2001–11	323.8±27.8	0	0	0	10.2±4.9	0.2±0.7	1079±50.5	0.22±0.7
Komi Republic	2000–11	144±3.8	0	0.01±0	0.1±0.1	62.7±2.5	0.6±0.2	431±6.6	0.16±0.1
Yamalo-Nenets AO	2001–11	143.1±5.2	0	1.59±0.6	0	42.3±2.8	2.58±0.7	414.8±8.9	0.24±0.2
Khanty-Mansi AO	2001–11	125.2±2.9	0.15±0.1	0.38±0.2	0.37±0.16	54.3±1.9	3.39±0.5	191.9±3.6	0.22±0.1
Taymyr AO	2001–06	100±16.2	0	1.14±1.7	nd	26.6±8.3	0.42±1.1	655.5±41.3	0
Evenki AO	2001–06	98.7±23.9	0	0	nd	244.9±37.5	0	476.4±52.3	0
Sakha Republic	2000–11	26.7±1.7	0	2.66±0.5	0.07±0.09	8.6±1	0.09±0.1	282.8±5.4	0.31±0.2
Magadan Oblast	2000–11	361±14.4	0	0	0	25.5±3.8	0.24±0.4	193.2±10.5	2.84±1.3
Koryak AO	2001–06	9.8±6.5	0	0	nd	31.2±11.6	0	812.5±58.7	0
Chukotka AO	2001–11	25.8±7	0	0	0	19.6±6.1	0.77±1.2	394.8±27.5	4.78±3
Kamchatka Kraj	2000–11	19±2.3	0	0.05±0.1	0	27.5±2.8	0.12±0.2	190.3±7.4	0.85±0.5
Sakhalin Oblast	2000–11	54.8±3.2	0	0.06±0.1	0	118.3±4.7	0.62±0.3	299±7.5	0.29±0.2
Khabarovsk Kraj	2000–11	84.1±2.4	0	0.05±0.1	0	24.8±1.3	0.11±0.1	200.3±3.8	0.77±0.2
Primorsky Kraj	2000–11	118.6±2.4	0	0.09±0.1	0	117.3±2.4	0.25±0.1	150.3±2.7	0.87±0.2
Amur Oblast	2000–11	38.5±2.1	0	0.05±0.1	0	50.9±2.4	0.66±0.3	358.4±6.4	0.74±0.3

Average incidence per 100,000 per year.

Giardiasis – *Giardia lamblia* (A07.1).

Cryptosporidiosis – *Cryptosporidium* sp. (A07.2). Data 2006–11.

Toxoplasmosis – *Toxoplasma gondii* (B58).

Amoebiasis – *Amoeba histolytica* (A06). Data 2009–11.

Ascariasis – (B77).

Trichuriasis – (B79).

Enterobiasis – (B80).

Trichinellosis – (B75).

Specified food- and waterborne helminthiases (laboratory-verified) are presented in Tables III and IV. Among *helminthiases* in Russia (2002–2011), enterobiasis (79%) and ascariasis (10%) predominate. In most of the studied regions, the most frequently occurring conditions are enterobiasis and ascariasis. The exceptions are Karelia Republic, Nenets AO and Sakha Republic (diphyllobothriasis in second place); Yamalo-Nenets AO (opisthorchiasis is in second place), and Khanty-Mansi AO (opisthorchiasis and enterobiasis).

**Table IV:** Reported food- and waterborne helminthiasis

Helminthiases									

	Years	Toxocariasis	Beef tapeworm	Pork tapeworm	Hymenolepiasis	Diphyllobothriasis	Echinococcosis	Opisthorchiasis	Clonorchiasis
Russian Federation	2002–11	1.58±0	0.38±0	0.13±0	0.5±0	10.18±0.1	0.37±0	26.77±0.2	0.17±0.01
Murmansk Oblast	2000–11	0.06±0.1	0.04±0.1	0.12±0.1	0.14±0.1	2±0.5	0.04±0.1	0.04±0.1	0
Karelia Republic	2000–11	1.44±0.5	0	0.04±0.1	0.09±0.1	31.7±2.1	0.05±0.1	0.06±0.1	0
Arkhangelsk Oblast	2000–11	0.44±0.2	0.01±0	0.02±0	0.07±0.1	5.5±0.7	0.27±0.1	0.25±0.1	0
Nenets AO	2001–11	0	1.08±1.6	1.5±1.9	0.65±1.2	213.9±22.6	1.07±1.6	0.87±1.4	0
Komi Republic	2000–11	0.21±0.1	1.35±0.4	1.17±0.3	0.24±0.2	37.8±2	0.11±0.1	50.31±2.3	0
Yamalo-Nenets AO	2001–11	4.41±0.9	9.28±1.3	0.31±0.2	0.84±0.4	87.5±4.1	5.62±1	409.7±8.8	0.06±0.11
Khanty-Mansi AO	2001–11	2.56±0.4	0.33±0.1	0.32±0.1	0.53±0.2	38.2±1.6	0.18±0.1	812.11±7.4	0.09±0.08
Taymyr AO	2001–06	0	0.38±1	2.4±2.5	0	248.7±25.5	0.76±1.4	6.58±4.1	nd
Evenki AO	2001–06	1.88±3.3	0.95±2.3	0	0	494.6±53.3	0.95±2.3	14.65±9.2	nd
Sakha Republic	2000–11	0.26±0.2	0.34±0.2	0.07±0.1	0.3±0.2	246.9±5.1	1.21±0.4	0.81±0.3	0
Magadan Oblast	2000–11	0.17±0.3	0	0.16±0.3	0	0.3±0.4	0.24±0.4	0.3±0.4	0
Koryak AO	2001–06	0	0	0	1.13±2.2	0	0.57±1.6	0	nd
Chukotka AO	2001–11	0.18±0.6	0.18±0.6	0	0.19±0.6	8.57±4.1	11.55±4.7	2.42±2.2	0
Kamchatka Kraj	2000–11	0.24±0.3	0.02±0.1	0.02±0.1	0.12±0.2	0.85±0.5	0.05±0.1	0.18±0.2	0
Sakhalin Oblast	2000–11	2.8±0.7	0.06±0.1	0	0.15±0.2	7.27±1.2	0.18±0.2	0.21±0.2	0.06±0.11
Khabarovsk Kraj	2000–11	2.16±0.4	0.07±0.1	0.12±0.1	0.17±0.1	1.93±0.4	0.24±0.1	0.36±0.2	0.84±0.24
Primorsky Kraj	2000–11	2±0.3	0.04±0	0.06±0.1	0.22±0.1	0.92±0.2	0.07±0.1	0.33±0.1	1.25±0.25
Amur Oblast	2000–11	0.45±0.2	0.1±0.1	0.1±0.1	0.71±0.3	0.98±0.3	0.22±0.2	0.98±0.3	21.24±1.55

Average incidence per 100,000 per year.

Toxocariasis – visceral larva migrans (B83.0).

Beef tapeworm – *Taenia saginata* (B68.1).

Pork tapeworm – *Taenia solium* (B68.0).

Hymenolepiasis – (B71.0).

Diphyllobothriasis – (B70.0).

Echinococcosis – (B67).

Opisthorchiasis – (B66.0).

Clonorchiasis – (B66.1). Data 2009–11.

*Ascariasis* is the typical endemic helminthiasis for a majority of selected regions. In Sakhalin and Primorsky Kraj, the rate of ascariasis is 3 times greater than in Russia as a whole, and in Evenki AO, the corresponding rate is 6 times higher than the national rate. The *Enterobiasis* rate is very high (twice or thrice the Russian national rate) in Arkhangelsk Oblast, Taymir, Korjak AO and Nenets AO. The average incidence of *Trichuriasis* (2000–2011) was about twice the average Russian rate in only 2 regions, Khanty-Mansi AO (545 cases totally), and Yamalo-Nenets AO (147 cases). Of 3,890 cases of *Trichinellosis* registered in the Russian Federation (2000–2011), 691 cases were registered in the selected regions, with the highest levels reported from regions in the Far East (135 cases in Khabarovsky Kraj, 219 cases in Primorsky Kraj and 83 in Amur Oblast). The regions with the highest rates (2.8–4.8 per 100,000) are Magadan Oblast (62 cases) and Chukotka (28 cases).

A growing problem, especially in the cities, is the increase of *toxocariasis*. This is a result of a significant increase in the number of dogs, coupled with the lack of disinfection of dog faeces in the cities, which leads to a high level of circulation of the pathogen in the urban environment. The highest levels of toxocariasis was registered in Yamalo-Nenets AO (258 cases), Khanty-Mansi AO (415 cases), Khabarovsky Kraj (367 cases) and Primorsky Kraj (491 cases). Of 6,667 cases of *T. saginata* registered in Russia (2000–2011), 843 cases were registered in the selected regions. The highest levels were reported in the Komi Republic (166 cases), Yamalo-Nenets AO (532 cases) and Khanty-Mansi AO (52 cases). Of 2,147 cases of *Taenia solium* taeniasis registered in Russia (2000–2011), 315 cases were registered in selected regions. The highest levels were reported from the Komi Republic (154 cases), Khanty-Mansi AO (53 cases) and Khabarovsk Kraj (20 cases). The regions with the highest incidences (1.2–2.4 per 100,000) are Nenets AO (7 cases) and Taymir (6 cases). *Hymenolepiasis* levels in the selected regions are generally comparable to the Russian national rate. *Diphyllobothriasis* is one of four main endemic helminthiases for many of the selected regions. In Nenets AO, Taymir and Yakutia, the rate of Diphyllobothriasis is 20 times greater than in Russia, and 50 times greater in Evenki AO. Of 6,388 cases of *Echinococcosis* registered in Russia (2000–2011), 775 cases were registered in the selected regions, with the highest levels reported from Yamalo-Nenets AO (336 cases), Yakutia (140 cases) and Chukotka (93 cases). *Opisthorchiasis* is a great problem mainly for 3 of the selected regions. These are the Komi Republic (6,141 cases in 2000–2011), Yamalo-Nenets AO (23,519 cases), and especially, Khanty-Mansi AO (130,679 cases). Uncontrolled transportation and storage of fish, lack of monitoring of technological methods of disinfecting the fish from *O. felineus* larvae, and intensive migration of the population cause deterioration of the epidemiological situation regarding opisthorchiasis in western Siberia, and in the whole of Russia. *Clonorchiasis* is widely spread in China, Japan and the Korean peninsula. In Russia, clonorchiasis is endemic in the Amur River basin where extremely high levels of this helminthiasis were registered. Of 719 cases of clonorchiasis registered in Russia (2009–2011), 659 cases were registered in the Far Eastern regions, – among them 35 cases in Khabarovsk Kraj, 74 in Primorsky Kraj, and 544 cases in Amur Oblast, which is 125 times higher compared to Russia, as a whole.

According to the current monitoring system, during 2000–2011 in the Russian Federation, 127 cases of *Anthrax* were registered, but no cases were registered in the selected regions. Animals spread this infection, and although not a conventional food- and water borne infection, it is an infection that causes great concern and is associated with the high level of mortality. The total number of *Cholera* cases for the same period in Russia was 11 (the majority of which were “imported” from abroad). In 2006, one case was detected in Murmansk Oblast as coming from India.

The internet site of *Rospotrebnadzor* (12), the owner of the official statistics includes the following information on *botulism*: “in the Russian Federation, since 2007 there have been about 200 cases of botulism a year, with the number of affected persons registered as about 300 per year.” An increase in the number of deaths has been observed in 15 deaths from botulism in 2007 to 26 deaths in 2010. Botulism in Amur Oblast was registered in 2000–2001 (7 women and 1 man, with one of the women dying) and occurred due to consumption of homemade dried low-salted pike and homemade vegetable squash (13). Generally, systematized data on botulism in the Russian regions are unavailable in statistics, though some information on several of the selected regions could be collected from the reports on sanitary-epidemiological situation (14–17). In 2006, 2 cases were registered in the Komi Republic (1 death); in 1992–2011 in Khanty-Mansi AO, there were 41 cases (no deaths). In 2004–2009, in Kamchatka Oblast, 16 cases were registered (1 death), and in 2009 in Sakhalin Oblast, 1 case was registered (no deaths).

## Discussion

The analysis of official statistical data on 18 selected regions of northern Russia as Russian Arctic, Siberia and the Far East (for the period 2000–2011) of infectious and parasitic food- and waterborne diseases has revealed alarming findings.

Among bacterial infections, a huge outbreak of typhoid in Yakutia in 2002, a high frequency of salmonella infections compared to the national average, a high excess of *S. sonnei* in several regions, a generally very high incidence of *S. flexneri* dysentery, and extremely high levels of *Y. enterocolitica* in Arkhangelsk Oblast and Sakhalin have been observed. Furthermore, infections with campylobacter have not been registered in 10 out of 18 regions.

Among viral infections, rotaviral enteritis frequently occurred in 8 regions. Norwalk gastroenteropathy levels were very different. In some regions the rates were very high. Hepatitis A rates and increasing tendencies are alarming; very high levels (50–100 cases per 100,000) have been reported from several regions. Among zoonotic infections, tularemia, brucellosis and leptospirosis were registered in the majority of selected regions. Giardiasis is the most common and widely spread among protozoa infections in selected regions and toxoplasmosis was registered in the majority of the regions selected.

Among helminthiases, enterobiasis, ascariasis, diphyllobothriasis, opisthorchiasis are endemic and very high in selected regions. Trichuriasis, toxocariasis, *T*. taeniasis and *T. solium* taeniasis levels were registered in a majority of regions, with high incidences in some regions. Echinococcosis was ubiquitous, and in some regions the levels were high. Trichinellosis rates in some regions were also very high. Clonorchiasis is endemic in the Amur River basin with extremely high levels registered.

Unequal numbers of sampling occasions in different Russian regions mean that data should be interpreted with caution, when comparing different regions. However, despite these defects in methodology, official Russian statistical data clearly reveal an acute situation from a public health perspective. For an international collaboration on the influence of climate change on infectious diseases, it is essential to monitor and compare international statistical data. Reports on Russian data are now annual, and this study containing data from the northern areas presents data of importance for international monitoring. One weakness is that not all infections have laboratory verifications.

## Conclusion

Both the incidence of infectious and parasitic food- and waterborne diseases among the general population of selected regions of Russian Arctic, Siberia and the Far East (in 2000–2011) is alarming, often exceeding the national incidences. The solutions must include a number of actions, such as a radical improvement of sanitary conditions of cities and settlements in the regions, cleaning of territories, construction of modern reliable water supply and sewage water systems, provision and strict control of high-quality drinking water and food, a full-scale reform of the general healthcare system and the monitoring system, enhancement of laboratory diagnostics and medical examination of populations, and the introduction of comprehensive prophylactic measures.

## References

[1] WHO Water and sanitation: facts and figures. http://www.euro.who.int/en/what-we-do/health-topics/environment-and-health/water-and-sanitation/facts-and-figures.

[2] Nilsson LM, Berner J, Dudarev AA, Mulvad G, Odland JO, Parkinson A (2013). Indicators of food and water security in an Arctic Health context – results from an international workshop discussion. Int J Circumpolar Health.

[3] Onishchenko GG (2010). On the state and measures to ensure the safety of drinking water in the Russian Federation. Hyg Sanit.

[4] Golovina SM, Rogovina AG (2008). Peculiarities of health status of population of Northern territories of Russian Federation. Probl Soc Hyg Hist Med.

[5] Vorobjev MV (2012). Incidence of viral hepatitis in the Russian Federation in 2009–2011. Soc Aspect Public Health.

[6] Vasilovsky AM, Kurkatov SV (2012). Hygiene assessment of influence of microbial and chemical contamination of food products on public health in Krasnoyarsk territory. Popul Health Environ.

[7] Schelchkova MV, Yadrikhinskaya VK (2008). Analysis of acute intestinal infectious diseases in population of Sakha republic (Yakutia) and Yakutsk city. Yakutian Med J.

[8] Ivanova LG, Nikiforov OI, Cherniavsky VF, Antonov NA (2007). About status of incidence and detection of giardiasis in Republic of Sakha (Yakutia). Yakutsk Med J.

[9] Miretsky GI, Dudarev AA (2004). The demographic situation and health status of indigenous peoples in the project study areas // Chapter 8 in the AMAP 2004: Persistent Toxic Substances, Food Security and Indigenous Peoples of the Russian North. Final Report.

[10] Onishchenko GG, Cherepov VM (2000). On sanitary-epidemiological wellbeing in eastern and western Siberia and about measures on its stabilization adopted in the framework of association “Siberian agreement”. Health Care Russ Federation.

[11] Egorov IJ, Kalinovsky AI, Maramovich AS, Cherniavsky VF (1997). Problems of epidemiological surveillance on brucellosis in conditions of northern reindeer herding. Epidemiol Infect Dis.

[12] Rospotrebnadzor Official website.

[13] Figurnov VA, Katin IS (2002). Botulism cases in the Amur region. Gig Sanit.

[14] (2007). On sanitary-epidemiological situation in Komi Republic, 2006: state report/Rospotrebnadzor.

[15] (2012). On sanitary-epidemiological situation in Khanty-Mansi AO, 2011: state report/Rospotrebnadzor.

[16] On sanitary-epidemiological situation in Kamchatka Kraj, 2009: state report/Rospotrebnadzor (2010).

[17] On sanitary-epidemiological situation in Sakhalin Oblast – 2009: state report/Rospotrebnadzor (2010).

